# Inhibitory gain modulation of defense behaviors by zona incerta

**DOI:** 10.1038/s41467-018-03581-6

**Published:** 2018-03-20

**Authors:** Xiao-lin Chou, Xiyue Wang, Zheng-gang Zhang, Li Shen, Brian Zingg, Junxiang Huang, Wen Zhong, Lukas Mesik, Li I. Zhang, Huizhong Whit Tao

**Affiliations:** 1 Zilkha Neurogenetic Institute, University of Southern California, Los Angeles, CA 90089 USA; 2 Graduate Program in Neuroscience, University of Southern California, Los Angeles, CA 90089 USA; 3 Department of Physiology, School of Basic Medical Sciences, Southern Medical University, Guangzhou, 510515 China; 4 Graduate Program in Biomedical and Biological Sciences, University of Southern California, Los Angeles, CA 90089 USA; 5 Department of Physiology and Neuroscience, University of Southern California, 1501 San Pablo Street, Los Angeles, CA 90089 USA

## Abstract

Zona incerta (ZI) is a functionally mysterious subthalamic nucleus containing mostly inhibitory neurons. Here, we discover that GABAergic neurons in the rostral sector of ZI (ZIr) directly innervate excitatory but not inhibitory neurons in the dorsolateral and ventrolateral compartments of periaqueductal gray (PAG), which can drive flight and freezing behaviors respectively. Optogenetic activation of ZIr neurons or their projections to PAG reduces both sound-induced innate flight response and conditioned freezing response, while optogenetic suppression of these neurons enhances these defensive behaviors, likely through a mechanism of gain modulation. ZIr activity progressively increases during extinction of conditioned freezing response, and suppressing ZIr activity impairs the expression of fear extinction. Furthermore, ZIr is innervated by the medial prefrontal cortex (mPFC), and silencing mPFC prevents the increase of ZIr activity during extinction and the expression of fear extinction. Together, our results suggest that ZIr is engaged in modulating defense behaviors.

## Abstract (web summary)

Zona incerta (ZI) is an inhibitory subthalamic nucleus with diverse connectivity yet its functional importance has not been extensively studied. Here the authors report that ZI receives mPFC input and can modulate both innate and learned defensive behaviors via its inhibitory projection to the PAG.

## Introduction

Zona incerta (ZI), first described more than a century ago by Auguste Forel^1^ as a “zone of uncertainty”, is a major subthalamic structure, functions of which remain largely unclear. Recently, it has become a region of interest and studies have revealed some important features of this region^2–5^. First, ZI has extensive efferent and afferent projections in connection with almost the entire neuroaxis, from cerebral cortices to the spinal cord^6–9^. This widespread connectivity may allow ZI to be involved in various physiological functions, such as feeding, sleeping, sensory-motor integration, maintenance of posture and locomotion, as well as regulation of pain^3–5,10–12^. ZI is also a clinically relevant structure since it has been implicated in alleviating symptoms of Parkinson’s disease by deep brain stimulation^13–16^. These findings raise an interesting hypothesis that ZI can serve as an important hub to coordinate and modulate various behaviors. The roles of ZI in different physiological and behavioral functions remain to be extensively explored.

ZI consists of heterogenous groups of cells, cytoarchitecture of which loosely divide the structure into multiple sectors^7,17,18^. In rodents, four sectors (rostral, ventral, dorsal, and caudal) of ZI can be defined based on the neurochemical expression pattern^19^. Given the various functional roles of ZI mentioned above, it would be interesting to investigate whether these different sectors might contribute to different aspects of ZI function.

In this study, we find that GABAergic neurons in the rostral sector of ZI (ZIr) project to the periaqueductal gray (PAG) in the midbrain. Many previous studies have suggested that PAG is an important commanding center to produce various types of defense behaviors^20–22^. We thus test whether ZIr activity could modulate these behaviors. Using optogenetic methods, we find that activation and suppression of ZIr reduces and enhances both innate and learned defensive behaviors respectively. Consistent with these behavioral effects, we find that ZIr directly inhibits excitatory neurons in both the dorsolateral and ventrolateral compartments of PAG. In addition, we provide evidence that ZIr is involved in extinction of conditioned fear response via the medial prefrontal cortex (mPFC)-ZIr connection. Together, our data suggest that ZIr plays a role in modulating defense behaviors based on experience or contexts.

## Results

### GABAergic ZIr projections to PAG

**Fig. 1:**
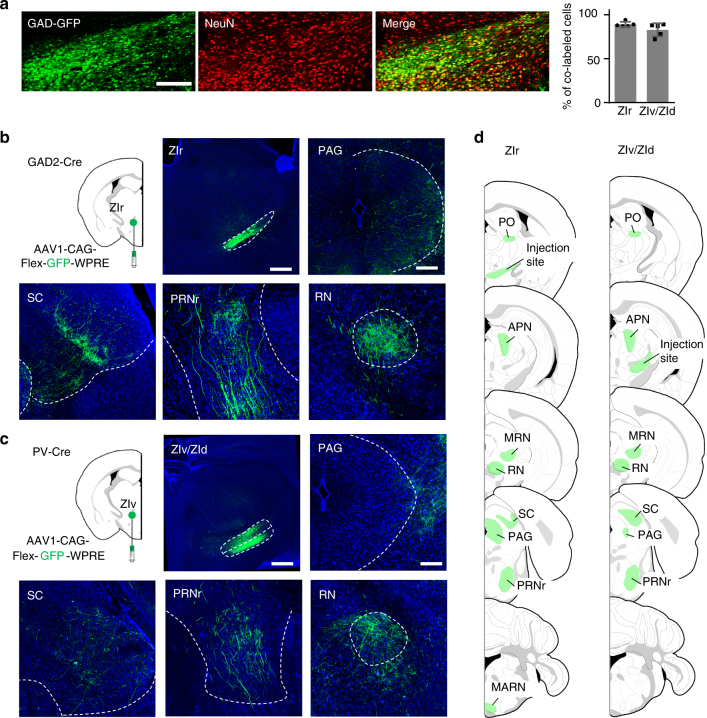
A distinct GABAergic projection from ZIr to PAG. **a** Left three panels, co-localization of GFP (green) signal and NeuN (red) staining in the ZIr of a GAD67-GFP mouse. Scale: 200 µm. Right panel, percentage of GFP^+^ neurons in the rostral sector (ZIr) and more caudal part (ZIv/ZId) of ZI. **b** Injection of Cre-dependent GFP virus into ZIr of GAD2-Cre mice. Confocal images show GFP expression in the injection site (upper middle; scale: 500 µm) and in several target structures (upper right and lower; scale: 200 µm). Blue shows Nissl staining. ZIr zona incerta, rostral, PAG periaqueductal gray, SC superior colliculus, PRNr pontine reticular nucleus, rostral, RN red nucleus. **c** Injection of Cre-dependent GFP virus into ZIv/ZId of PV-Cre mice. Images show GFP expression in the injection site (upper middle; scale: 500 µm) and in several target structures (upper right and lower; scale: 200 µm). **d** Summary of target areas of GAD2^+^ ZIr (left panel) and PV^+^ ZIv/ZId (right panel) neurons. PO posterior complex of the thalamus, APN anterior pretectal nucleus, MRN midbrain reticular nucleus, MARN magnocellular reticular nucleus

Although ZI is known as an inhibitory nucleus^2,23^, diverse cell types have been reported in this structure^19,24,25^. We first sought to understand the proportion of GABAergic neurons within the ZI. For this, slices from the GAD67-GFP mouse, in which all GABAergic cells are labeled with GFP^26^, were stained with NeuN to label neuronal cell bodies. Consistent with previous reports^2,3,5^, we found that the majority of neurons in ZI, in particular the rostral part of ZI (ZIr), were GABAergic cells (Fig. 1a). To understand where these neurons project to, we performed focal injections of adeno-associated virus (AAV) encoding Cre-dependent GFP into inhibitory neuron specific Cre lines. GAD2, a pan GABAergic cell marker, is expressed throughout the ZI^19^, but in GAD2-Cre mice we limited our injections in ZIr (Supplementary Fig. 1a, inset, and see Methods). Parvalbumin (PV) is most strongly expressed in the ventral sector of ZI (ZIv) and less in its dorsal sector (ZId)^2^. Accordingly, in PV-Cre mice we made injections in the more caudal part of ZI (Methods). Comparison of efferent projection patterns from the injections in these two different Cre lines revealed a clear difference. For the ZIr injection in GAD2-Cre mice, profuse GFP-labeled axons were found in PAG, including both its dorsolateral and ventrolateral compartments (dlPAG and vlPAG respectively) (Fig. 1b), consistent with previous observations^27,28^. In contrast, there were few axonal projections to PAG for the ZIv/ZId injection in PV-Cre mice (Fig. 1c). Other than this, the ZIr and ZIv/ZId injections revealed similar axonal labeling patterns in the midbrain (such as in SC, RN, and MRN), hindbrain (such as PRN), and thalamus (such as PO) (Fig. 1d and Supplementary Fig. 1a, b), while few projections were found in cortical regions or the amygdaloid complex (Fig. 1d). Retrograde labeling using rabies virus confirmed that most PAG-projecting ZI neurons were located in ZIr (Supplementary Fig. 1c, d). From these results, we have identified a distinct GABAergic projection from ZIr to PAG.

### ZIr bidirectionally modulates innate flight response

**Fig. 2:**
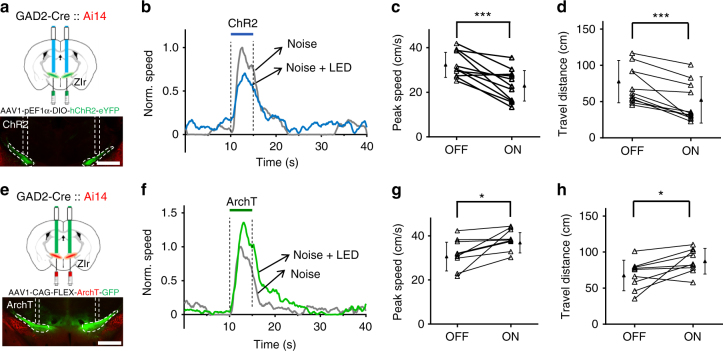
ZIr bi-directionally modulates noise-induced flight response. **a** Upper, illustration of the experimental paradigm. Lower, image showing ChR2 expression (green) and placement of two optic fibers over ZIr. Scale: 1000 µm. **b** Normalized average speed induced by noise sound for an example ChR2-expressing animal with (blue) and without (gray) blue LED stimulation. Two dash lines mark the duration of noise. **c** Summary of noise-induced peak speed without (OFF) and with (ON) activation of ZIr neurons. ***p = 0.001, two-sided paired *t*-test, *n* = 11 animals. **d** Summary of total travel distance during the 5-s noise stimulation without and with activation of ZIr neurons. ***p = 0.003, two-sided Wilcoxon signed-rank test, *n* = 11. **e** Upper, illustration of the experimental paradigm. Lower, image showing ArchT expression (green) and placement of two optic fibers over ZIr. Scale: 1000 µm. **f** Normalized average speed induced by noise for an example ArchT-expressing animal with (green) and without (gray) green LED stimulation. **g** Summary of noise-induced peak speed without and with suppressing ZIr neurons. *p = 0.019, two-sided paired *t*-test, *n* = 9 animals. **h** Summary of total travel distance without and with suppressing ZIr. *p = 0.032, two-sided paired *t*-test, *n* = 9 animals. Solid symbol represents mean ± s.d. for all panels

PAG is known to be a commanding center to produce various types of defense behaviors^20–22^, and previous studies have suggested that dlPAG and vlPAG drive flight and freezing types of defense behaviors respectively^20,21^. Since both dlPAG and vlPAG receive GABAergic projections from ZIr, we speculated that ZIr might be able to modulate both flight and freezing types of PAG-mediated behaviors^29^. Previously, we have reported that loud noise can trigger flight response in naïve freely moving or head-fixed mice, as manifested by a robust increase of their running speed, and that this behavior is mediated by dlPAG^20^. We first tested whether ZIr could modulate this innate flight behavior in head-fixed mice (Methods). To this end, we optogenetically targeted GABAergic neurons in ZIr by focal injections of AAV encoding Cre-dependent channelrhodopsin2 (ChR2) for activation or ArchT for suppression^30,31^ in GAD2-Cre mice (Supplementary Fig. 2a, b). Blue or green LED light was delivered bilaterally through implanted optic fibers (Fig. 2a, e). LED light was applied during noise stimulation (80 dB sound pressure level) in trials interleaved with control LED off trials. We found that both the peak running speed and travel distance of noise-induced running were significantly reduced in trials when ZIr neurons were activated as compared with control trials (Fig. 2b–d). Opposite effects were observed when ZIr neurons were suppressed: the peak speed and travel distance both increased (Fig. 2f–h). Changes in running were not observed in GFP-expressing control animals (Supplementary Fig. 2c–e). Despite the changes in behavioral amplitude, the onset latency of the flight response was not affected by either type of optogenetic manipulation (Supplementary Fig. 2f, g). The LED stimulation alone, either with blue or green light, did not change the baseline locomotion in the absence of noise stimulation, or balance beam performance (Supplementary Fig. 3). Together, these results demonstrate that ZIr activity can bidirectionally modulate the magnitude of innate flight response.

### ZIr exerts a gain modulation function

**Fig. 3:**
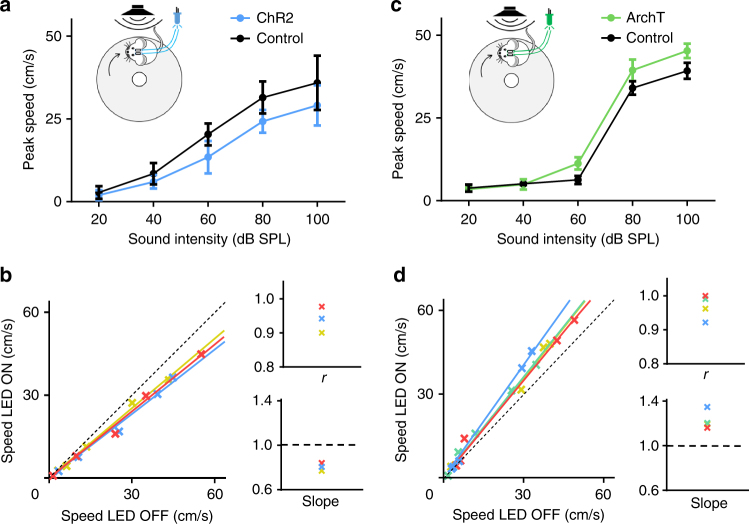
ZIr activity exerts a gain modulation. **a** Average peak speeds induced by noise of different intensities without (black) and with (blue) activation of ZIr neurons for an example animal. Inset, illustration of experimental setup. **b** Left, peak speed in the LED on condition versus that in the LED off condition plotted for three animals. Each color represents one animal. Colored lines represent the corresponding linear regression line. Black dash line is the unity line. Right, correlation coefficient calculated for each animal (upper) and the slope for the best-fit linear regression line (lower). **c** Average peak speeds induced by noise of different intensities without (black) and with (green) suppression of ZIr neurons for an example animal. **d** Speed with ZIr suppression versus that without suppression plotted for four animals

As having been reported^20^, the magnitude of innate flight response increases with increasing noise intensities. In a separate cohort of animals, we examined how ZIr activity affected running speeds under different noise intensities (Fig 3a, c, black). We found that optical manipulations of ZIr activity using ChR2 and ArchT reduced and enhanced respectively flight responses across effective noise intensities (Fig. 3a, c, color). Importantly, the peak running speed in the LED on condition was linearly correlated with that in the LED off condition in each examined animal (Fig. 3b, d). These data demonstrate that the magnitude of flight response under different sound intensities is divisively modulated by changing ZIr activity. Specifically, under our current optical stimulation condition (5 mW on one side), activation of ZIr reduced the flight speeds across different noise intensities by ~20%, whereas suppression of ZIr increased the flight speeds by ~20%. Our data thus support the notion that ZIr can exert a gain control function in modulating innate flight response.

### ZIr bidirectionally modulates conditioned freezing response

**Fig. 4:**
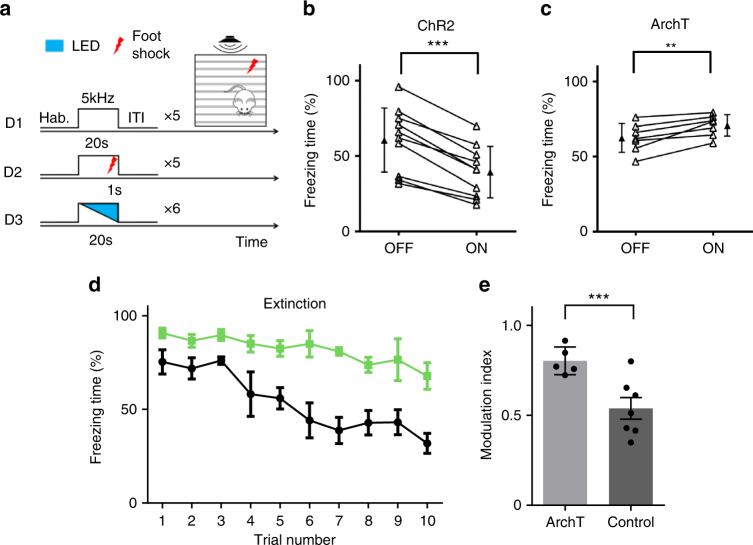
ZIr bidirectionally modulates conditioned freezing response. **a** Experimental paradigm for fear conditioning and testing of learned freezing response. **b** Percentage of time freezing during presentations of CS alone without and with activation of ZIr. ***p < 0.001, two-sided paired* t*-test, *n* = 10 animals. **c** Percentage of time freezing during presentations of CS alone without and with suppressing ZIr. **p = 0.005, two-sided paired *t*-test, *n* = 7 animals. **d** Percentage of time freezing at each presentation of CS during extinction training with (green) and without (black) suppressing ZIr. Scale bar = s.e., *n* = 5 for ArchT and 7 for control. **e** Modulation index calculated for each animal (as the ratio of freezing time between the last and first trial) for the GFP control and ArchT suppression group. ***p = 0.004, two-sided unpaired *t*-test, *n* = 5 for ArchT and 7 for control. Solid symbol represents mean ± s.d. for all panels

Since vlPAG has been implicated in mediating conditioned freezing response^21^, we reasoned that ZIr might be able to modulate this behavior as well. To test this possibility, we applied similar optogenetic approaches. The animal was first exposed to a conditioned stimulus (CS, 20-s 5 kHz tone) for five times in a test box during day 1 (Fig. 4a). In day 2, it was exposed to the CS paired with a foot shock (1 s) for five times in a conditioning chamber. In day 3, the CS was applied without a foot shock for six trials in the test box in order to measure cued conditioned freezing response. In half of these trials, blue or green LED stimulation was applied during the CS presentation in a randomized order. We found that freezing time during CS presentation was significantly reduced in trials when ZIr neurons were activated as compared with control trials (Fig. 4b). On the other hand, freezing time was increased when ZIr neurons were suppressed (Fig. 4c). Freezing time was not significantly changed by LED stimulation in GFP-expressing control mice (Supplementary Fig. 4a). These data demonstrate that increasing and decreasing ZIr activity can dampen and enhance conditioned freezing response, respectively. Therefore, ZIr is able to modulate bidirectionally the magnitude of both innate and learned defensive behaviors.

### ZIr is engaged in extinction of conditioned freezing

It is known that conditioned freezing response naturally diminishes with repeated presentations of CS alone, a process termed “extinction”^32,33^. In other words, learned fear response is naturally modulated in an experience-dependent manner. Since ZIr activation could reduce the freezing response, we wondered whether ZIr might be involved in its extinction. To test this possibility, we expressed ArchT in ZIr neurons using GAD2-Cre mice. We conditioned the mice similarly with five pairings of CS and foot shock. On the test day, the animal was presented with CS alone for ten trials in order to obtain an extinction curve^34,35^. In GFP-expressing control mice, freezing was significantly reduced after the first five trials (Fig. 4d, black), consistent with previous observations^34,35^. For the experimental group in which green LED light was paired with each CS presentation to suppress ZIr activity, the reduction of freezing time over repeated CS presentations was much slower (Fig. 4d, green), as shown by the quantification of a modulation index (Fig. 4e), indicating that extinction was impaired. In addition, one day following the normal extinction training, suppressing ZIr activity also impaired the expression of extinction retrieval^36,37^ (Supplementary Fig. 4b). These data further demonstrate that ZIr activity regulates the expression of conditioned fear response.

**Fig. 5:**
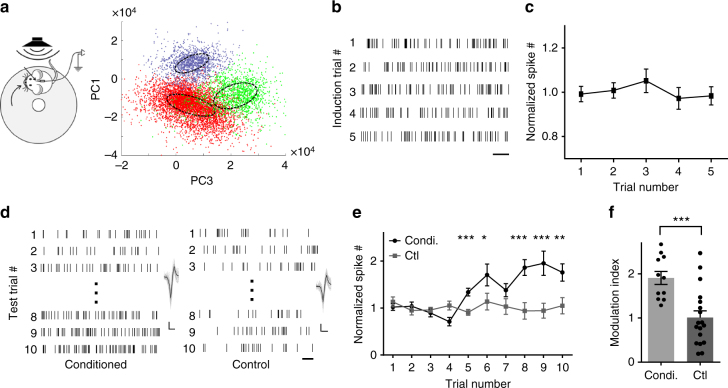
ZIr activities during fear conditioning and fear extinction. **a** Left, experimental setup for extracellular recording. Right, principal component analysis for an example recording session. The graph shows the projection of principal components to the PC1–PC3 plane. Colors mark different clusters of the spikes. Dashed circles denote the boundary of well separated spikes. **b** Raster plot of spikes for an example ZIr unit during CS presentation (19 s, just before the foot shock). Scale: 2 s. **c** Average normalized spike rate of ZIr units during fear induction, *n* = 12 units from four animals. **d** Raster plot of spikes for example units during the first and last three presentations of CS alone in an example conditioned and non-conditioned control mouse respectively. Scale: 0.5 s. Inset, superimposed 50 individual spikes and their average (black) for the corresponding unit. Scale: 20 µV, 0.5 ms. **e** Average normalized spike rate of ZIr units at each CS presentation in conditioned (black, *n* = 11 units from six animals) and control (gray, *n* = 18 units from six animals) mice. ***p(trial5) < 0.001, *p(trial6) = 0.031, ***p(trial8) = 0.003, ***p(trial9) < 0.001, **p(trial10) = 0.006, two-sided Mann–Whitney *U* test. **f** Modulation index calculated for each unit for the conditioned and control groups. ***p < 0.001, two-sided unpaired *t*-test. Solid symbol represents mean ± s.e.m. for all panels

We next monitored spiking activity in ZIr by performing in vivo single-unit recordings (Fig. 5a and Supplementary Fig. 5a, Methods). Animals were either conditioned similarly, or exposed to CS without foot shocks (control). Spikes of ZIr neurons were recorded during presentations of CS either on the conditioning day, or in the following day when conditioned freezing exhibited extinction over repeated CS presentations (Supplementary Fig. 5b, c). ZIr activity did not change significantly during conditioning, i.e., pairing of CS with foot shocks (Fig. 5b, c). Interestingly, in the following day, ZIr activity was found to increase progressively with repeated CS presentations in the conditioned but not control mice (Fig. 5d–f). This observed increase of ZIr activity is consistent with the above result that enhancing ZIr activity led to reduced freezing. These results suggest that ZIr may be naturally engaged in extinction.

**Fig. 6:**
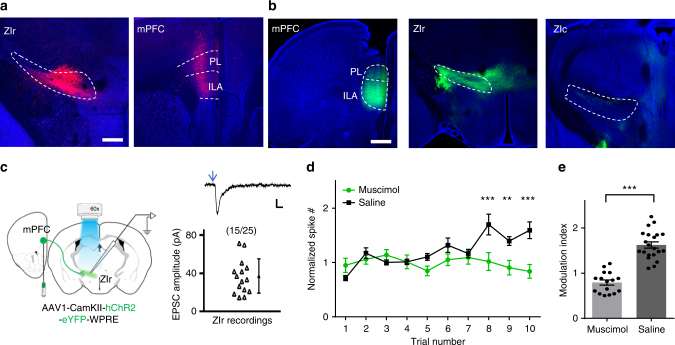
mPFC contributes to ZIr activity increase during extinction. **a** Injection of rAAV2-retro-syn-Cre into ZIr of Ai14 mouse. Left, tdTomato expression around the injection site. Right, retrogradely labeled presynaptic neurons (red) in mPFC. Blue shows Nissl staining. ILA and PL subregions are marked. Scale: 500 µm. **b** Injection of GFP virus into mPFC of wild-type mice. Left, GFP expression at the injection site. Middle, GFP-labeled axons in ZIr. Right, no GFP-labeled axons in the more caudal part of ZI. Scale: 500 µm. **c** Left, experimental paradigm. Right, average EPSC amplitudes of 15 out of 25 recorded ZIr neurons in response to LED activation of mPFC axons. Recording was made in the presence of TTX and 4-AP. Top inset, average EPSC trace for an example ZIr neuron. Arrow points to the onset of LED. Scale: 40 pA, 10 ms. **d** Average normalized spike rate of ZIr units during extinction training for saline control (black, *n* = 21 units from seven animals) and mPFC muscimol injected (green, *n* = 16 units from six animals) mice. ***p(trial 8) < 0.001, **p(trial9) = 0.002, ***p(trial10) < 0.001, two-sided Mann–Whitney *U* test. **e** Modulation index calculated for each unit in the saline and muscimol group. ***p < 0.001, two-sided unpaired *t*-test. Solid symbol represents mean ± s.e.m. for all panels

Previously the mPFC has been implicated in extinction^38,39^, and its projection to ZI has been reported^40,41^. We wondered whether mPFC might play a role in driving the activity increase in ZIr during extinction. We first performed retrograde labeling by injecting rAAV2-retro-syn-Cre into ZIr of Ai14 mice^42^ (Fig. 6a), and found robust labeling in mPFC subregions such as the infralimbic area (ILA), prelimbic area (PL), as well as part of the anterior cingulate area (ACA). To confirm the functional connectivity, we injected AAV-ChR2-eYFP into mPFC, mainly in ILA and PL subregions (Fig. 6b, left). Dense axonal projections were found in ZIr, but not in the more caudal part of ZI (Fig. 6b, middle and right). In brain slices, we performed whole-cell recordings and found that a majority of recorded ZIr cells exhibited monosynaptic excitatory responses to the optogenetic activation of mPFC axons (Fig. 6c), confirming the mPFC-ZIr connectivity. We next injected muscimol into mPFC to silence its local spiking activity (Supplementary Fig. 6a). This prevented the increase of ZIr activity during extinction, whereas the increase was still observed in saline-injected control animals (Fig. 6d, e). In addition, silencing mPFC with muscimol impaired extinction of freezing response behaviorally (Supplementary Fig. 6b-c). Our data thus suggest that the engagement of ZIr during extinction is mediated at least partially by the mPFC-ZIr projection.

### Inhibitory effects of ZIr input to PAG

**Fig. 7:**
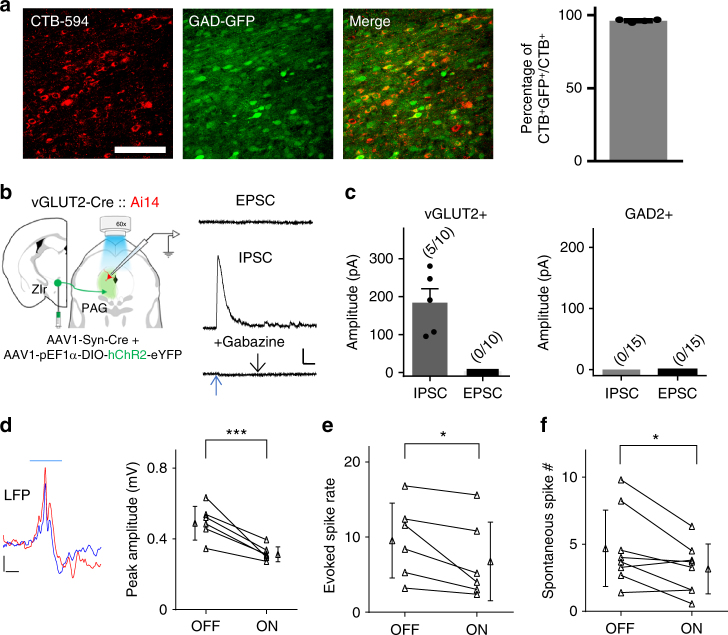
GABAergic output of ZIr suppresses PAG activity. **a** Left three panels, co-localization of GFP (green) with CTB (red) retrogradely transported from PAG in the ZIr of a GAD67-GFP mouse. Scale: 200 µm. Right panel, percentage of GAD-GFP^+^ neurons in total CTB labeled neurons in ZIr. **b** Left, slice recording paradigm. Excitatory neurons are labeled by red color in vGLTU2-Cre::Ai14 mice. Right, average EPSC (recorded at −70 mV) and IPSC (recorded at 0 mV) traces of a vGLUT2^+^ neuron in PAG. The IPSC was blocked by Gabazine (bottom). Blue arrow points to the onset of LED. Scale: 50 pA, 50 ms. **c** Average amplitudes of LED-evoked IPSCs and EPSCs in excitatory (left) and inhibitory (right) PAG neuron populations. **d** Left, average local field potential recorded in PAG in response to noise without (red) and with (blue) LED stimulation in a ZIr ChR2 expressing mouse. Right, summary of peak amplitude of LFP recorded in PAG without (OFF) and with (ON) activation of ZIr. ***p = 0.004, two-sided paired *t*-test, *n* = 6 sites from two animals. **e** Summary of noise-evoked spike rate in PAG without and with ZIr activation. *p = 0.028, two-sided Wilcoxon signed-rank test, *n* = 6 sites from two animals. **f** Summary of spontaneous firing rate in PAG without and with ZIr activation. *p = 0.03, two-sided paired *t*-test, *n* = 8 sites from two animals. Solid symbol represents mean ± s.d. for **d**, **e**,** f**, and mean ± s.e.m. for **a**,** c**

To further elucidate the nature of connectivity between ZIr and PAG, we injected a retrograde dye, CTB, into PAG of GAD67-GFP mice. Nearly all retrogradely labeled neurons in ZIr colocalized with GFP (Fig.7a), confirming that PAG-projecting ZIr neurons are GABAergic. In slice preparations, we made whole-cell recordings from PAG neurons, with TTX and 4AP present in the extracellular solution to block polysynaptic responses (Methods). To label excitatory or inhibitory PAG neurons, we used vGLUT2-Cre or GAD2-Cre crossed with Ai14 mice, respectively. We found that only excitatory but not inhibitory PAG neurons received monosynaptic inhibitory input from ZIr, which could be blocked by a GABA_A_ receptor blocker, Gabazine (Fig. 7b, c). This is the case for both dlPAG and vlPAG (Supplementary Fig. 7). On the other hand, none of the recorded neurons exhibited monosynaptic excitatory responses (Fig. 7b, c), as expected from the GABAergic cell type of ZIr neurons projecting to PAG. We further carried out multiunit and local field potential (LFP) recordings in PAG^20^ in vivo, and found that the peak amplitude of LFP and multiunit spike rate evoked by noise sound were reduced by optogenetic activation of ZIr (Fig. 7d, e). In addition, the spontaneous spike rate in PAG was also reduced by the activation of ZIr (Fig. 7f). All these data demonstrate that the ZIr to PAG projection is inhibitory and can directly suppress PAG output, which is consistent with the above results showing a suppressive effect of ZIr on PAG-mediated defense behaviors.

### ZIr modulates defense behavior via its projection to PAG

**Fig. 8:**
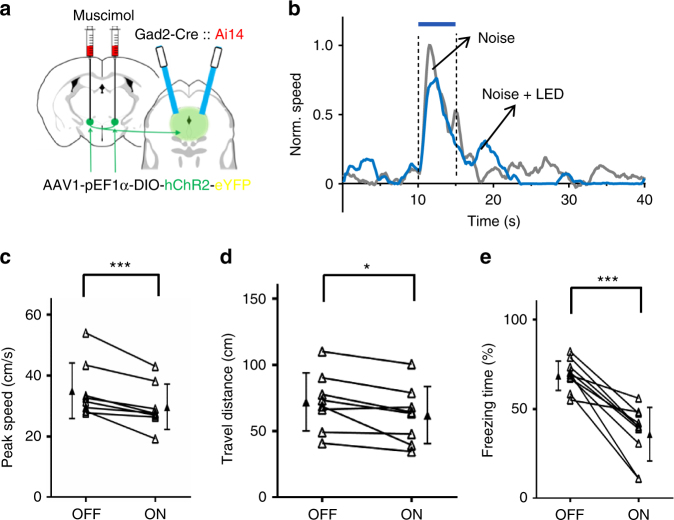
The ZIr-PAG projection mediates the modulatory effects on defense behaviors. **a** Experimental paradigm. LED was applied to PAG, and muscimol was injected into ZIr as to prevent antidromic stimulation. **b** Normalized average speed responding to noise without (gray) and with (blue) LED stimulation plotted for an example animal. **c** Summary of noise-induced peak speed without and with activation of ZIr-PAG projections. ***p = 0.003, two-sided paired *t-*test, *n* = 8 animals. **d** Summary of noise-induced travel distance without and with activation of ZIr-PAG projections. *p = 0.021, two-sided paired *t*-test, *n* = 8 animals. **e** Percentage of time freezing during presentations of CS alone without and with activation of ZIr-PAG projections in conditioned mice. ***p < 0.001, two-sided paired *t*-test, *n* = 10 animals. Solid symbol represents mean ± s.d. for all panel

Similar as reported previously^20^, optogenetic activation of dlPAG neurons directly induced flight response, and the magnitude of the induced response increased with increasing LED intensities (Supplementary Fig. 8). To further test whether ZIr regulates the magnitude of defense behaviors by directly modulating PAG activity, we optically activated ChR2-expressing ZIr axons in PAG bilaterally while silencing ZIr neuronal cell bodies with muscimol to prevent antidromic spikes (Fig. 8a). Results similar as activating ZIr cell bodies were obtained: the peak speed and travel distance of noise-induced flight response were significantly reduced as compared with control trials (Fig. 8b–d), and the conditioned fear response was also reduced (Fig. 8e). These results indicate that the inhibitory ZIr-PAG projection can mediate the modulatory effects of ZIr on both types of defense behaviors.

## Discussion

In this study, by using combined anatomical tracing, electrophysiological recording and bidirectional optogenetic manipulations, we demonstrate that the rostral part of ZI can bidirectionally modulate defense behaviors, including both innate and learned defensive behaviors. By testing flight responses under different sound intensities, we further demonstrate that the modulation by ZIr is likely through a gain control mechanism. Moreover, for a natural form of experience-dependent modulation of defense behavior, fear extinction, we demonstrate that ZIr activity increase is correlated with the expression of extinction, i.e., reduced freezing over repeated presentations of CS alone. This finding further supports the engagement of ZIr in modulating the level of defense behaviors based on experience. Finally, we provide evidence that the modulatory effects of ZIr are achieved through its inhibitory projection to PAG. Our results together have unveiled a novel function of ZIr in regulating defense behaviors.

ZI is a relatively large inhibitory subthalamic nucleus containing multiple sectors and cell types^7,17–19^. Previous studies have also demonstrated that ZI receives a rich variety of inputs from many brain regions, including multisensory inputs and neuromodulatory inputs that carry information about brain state^2,9,43,44^. This cytoarchitectural and connectivity complexity may be the basis for the ZI to be involved in various aspects of animals’ physiological and behavioral functions. For example, clinically, ZI has been implicated in alleviating symptoms of Parkinson’s disease as well as essential tremor by deep brain stimulation^13–16^. More recently, it has been reported that stimulation of ZI GABAergic neurons evokes binge-like eating, resulting in body weight gain^3^. A subpopulation of GABAergic neurons in the ventral ZI, the Lhx6-positive neuron, has also been shown to promote sleep^5^. In the current study, we have discovered a new functional role of ZIr in modulating defense behaviors. All these findings help us to better understand the overall function of ZI.

PAG is a commanding center responsible for initiating various defense behaviors^20–22^. Our data demonstrate that ZIr sends GABAergic projections to both dlPAG and vlPAG. This allows ZIr to be able to modulate diverse types of defense behavior. It is worth noting that other brain regions containing GABAergic neurons such as the hypothalamus also project to both compartments of PAG (Allen Brain Atlas). These areas may also be able to exert similar regulatory functions on defense behaviors. Besides PAG, ZI projects broadly to other motor-related midbrain and hindbrain structures such as the superior colliculus (SC), midbrain reticular nucleus (MRN), red nucleus (RN), and pontine reticular nucleus (PRN)^9,45,46^. The GABAergic projections of ZI to these areas may contribute to the regulation of locomotor activities and posture as well. It would be interesting to distinguish different cell types within ZI sectors and examine whether they have distinct projection patterns. Such information may provide insights into unique functional roles of subpopulations of ZI neurons.

Our anatomical results and data from online resources for connectome (Allen Brain Atlas) indicate that ZI receives input from mPFC subregions including ACA, PL, and ILA. It is thus reasonable to postulate that by bridging higher cortical areas and midbrain/hindbrain nuclei, ZI may serve as a regulatory hub to mediate the top-down regulation of various motor behaviors, in addition to that mediated by direct projections from cortical areas^28,40^. When the environment becomes safer or when tangible threats are removed, signals may be transmitted to ZI to reduce defense and promote eating or sleeping. Whether some other inhibitory nuclei can serve a similar function is worth further explorations.

It is an intriguing finding that the ZIr’s effect on defense behavior is through a gain modulation mechanism, i.e., ZIr modulates the amplitude of behavioral output. Such gain modulation may have a great advantage. In a natural environment, danger signals may vary greatly in strength, from mildly intimidating to life threatening, and there could be hidden unperceived threats. Maintaining certain levels of defense is protective and beneficial, while prolonged or intensified defense may cause a failure of the animal to adapt to the changing environment timely^47–49^. A gain control mechanism allows adjustment of the magnitude of ongoing defense behavior in accordance with the current danger level. In our experiments, the observed modulation of behavioral response was moderate. However, it is unknown what the upper limit of ZIr modulation could be, since the activation or suppression of ZIr neurons in our experiments was unlikely complete. It remains unclear how the gain modulation function of ZIr is achieved. Our data have implied that the level of spiking activity in its downstream structure, PAG, correlates with the magnitude of defense behavior (Supplementary Fig. 8). Does ZIr activation suppress spike rates of PAG neurons proportionally under different danger levels? This question awaits further investigations in the future.

We demonstrate that ZIr activity increase correlates with a reduction of conditioned fear response during extinction, which supports the notion that ZIr is naturally engaged in defense modulation. We have also identified mPFC as a potential input source that drives the engagement of ZIr. There have been a vast number of studies on the circuits underlying fear extinction^36,37^. Based on the observed effect of ZIr suppression on fear extinction, we postulate that the identified ZIr-PAG pathway could serve in parallel with the classic amygdala-PAG pathway^43,50^ to reduce fear expression during extinction. While it is generally accepted from previous studies that PL and ILA have opposite effects on fear expression^34,39^, it would be interesting to further investigate how each subregion of mPFC influences ZIr during extinction.

In summary, we have discovered a role of ZI, a major inhibitory subthalamic nucleus, in modulating defense behaviors. We propose that ZI can serve as a bridge between higher cortical areas and midbrain/hindbrain nuclei for the top-down regulation of motor behaviors. This inhibitory nucleus mediated gain modulation may be a general functional strategy implemented by mammalian brain circuits.

## Methods

### Animals

All experimental procedures used in this study were approved by the Animal Care and Use Committee at the University of Southern California. Male and female wild-type (C57BL/6) and transgenic (GAD2-Cre, vGLUT2-Cre, PV-Cre, and Ai14) mice aged 8–16 weeks were obtained from the Jackson Laboratory. Animal sample sizes were determined by the estimated variances of the experiments and previous experience from similar experiments, and were sufficient for all the statistical testes. Mice were housed on 12 h light/dark cycle, with food and water provided ad libitum. Randomization methods were used to allocate experimental groups.

### Viral and tracer injection

Viral injections were carried out as we previously described^51,52^. Stereotaxic coordinates were based on the Allen Reference Atlas (www.brain-map.org). Mice were anesthetized using 1.5% isoflurane throughout the surgery procedure. A small incision was made on the skin after shaving to expose the skull. A 0.2 mm craniotomy was made and virus was delivered through a pulled glass micropipette with beveled tip (~20 µm diameter) by pressure injection. For anterograde tracing, AAV1-CAG-FLEX-eGFP-WPRE-bGH (UPenn Vector Core, 1.7 × 10^13^ GC/ml) was injected into the rostral sector of ZI (30 nl total volume; AP −1.2 mm, ML +1.6 mm, DV −4.4 mm) of GAD2-Cre mice. The same virus was also injected into ZIv/ZId (30 nl total volume; AP −1.5 mm, ML +2.2 mm, DV −4.2 mm) of PV-Cre mice. Animals were euthanized 3–4 weeks following the injection for examination. For retrograde tracing, EnvA G-deleted Rabies-eGFP (Addgene# 32635) was injected into dlPAG (30 nl total volume; AP −4.4 mm, ML +0.6 mm, DV −2.4 mm) and vlPAG (30 nl total volume; AP −4.4 mm, ML +0.5 mm, DV −2.7 mm) of wild-type C57BL/6 mice, respectively. CTB-584 was injected into PAG (30 nl total volume; AP −4.4 mm, ML +0.5 mm, DV −2.5 mm). rAAV2-retro-syn-Cre (Vigene, custom order) was injected into ZIr (30 nl total volume; AP −1.2 mm, ML +1.6 mm, DV −4.4 mm) of Ai14 mice. Animals were sacrificed 1 week after the injection.

For behavioral and electrophysiological assessments, AAV2/1-pEF1α-DIO-hChR2-eYFP (UPenn Vector Core, 1.6 × 10^13^ GC/ml), AAV1-CAG-FLEX-ArchT-GFP (UNC Vector Core, 1.6 × 10^13^ GC/ml), or AAV1-CAG-FLEX-eGFP-WPRE-bGH (UPenn Vector Core, 1.7 × 10^13^ GC/ml, as control) was injected bilaterally into ZIr (100 nl for each site) of GAD2-Cre::Ai14 mice. For the vGLUT2-Cre::Ai14 mice, AAV1-Syn-Cre mixed with AAV2/1-pEF1α-DIO-hChR2-eYFP was injected bilaterally into ZIr (100 nl for each site). AAV1-CamKII-hChR2(E123A)-eYFP-WPRE-hGh (UPenn Vector Core, 1.6 × 10^13^ GC/ml) was injected into dlPAG or mPFC (30 nl total volume; AP +1.6 mm, ML −0.4 mm, DV −1.9 mm) of wild-type C57BL/6 mice. Viruses were expressed for at least 3 weeks.

### Histology and imaging and quantification

Animals were deeply anesthetized and transcardially perfused with phosphate buffered saline (PBS) followed by 4% paraformaldehyde. Brains were post fixed at 4 ˚C overnight in 4% paraformaldehyde and then sliced into 150 μm sections using a vibratome (Leica, VT1000s). To reveal the cytoarchitectural information, brain slices were first rinsed three times with PBS for 10 min, and then incubated in PBS containing Nissl (Neurotrace 620, ThermoFisher, N21483) and 0.1% Triton X-100 (Sigma-Aldrich) for 2 h. All images were acquired using a confocal microscope (Olympus FluoView FV1000). For NeuN staining, sections were blocked with 5% normal goat serum/0.05% Triton X-100 for 2 h at room temperature, then incubated with mouse IgG monoclonal anti-NeuN antibody (MAB377; Millipore, Billerica, MA) at 1:2000 overnight at 4 °C. Sections were washed three times in PBS for 10 min and exposed to a secondary anti-mouse IgG conjugated with Alexa fluor 549 (115-505-003; Jackson, Cambridge, MA) at 1:1000 for 2 h at room temperature. In quantification of retrograde labeling in ZI, cell bodies labeled with GFP were counted manually across all sections containing ZI, and the relative number of labeled cells in different compartments of ZI was calculated as the percentage of total labeled cells across ZI. The values were then averaged across animals. To obtain the anterograde projection patterns of ZIr or ZIv/ZId, serial sections across the whole brain were collected and imaged under ×4 objective. Regions with axonal labeling were then imaged under 10 × objective across the depth of the tissue (15 μm z-stack interval). Each image was taken using identical laser power, gain and offset values.

### Optogenetic preparation and stimulation

One week before the behavioral tests, animals were prepared as previously described^20^. Briefly, to optogenetically manipulate ZIr cell bodies or axon terminals, mice were implanted with fiber optic cannula (200 µm ID, Thorlabs) two weeks after injecting ChR2, ArchT or GFP virus^30,31^. The implantation was made while the animal was anaesthetized and mounted on stereotaxic apparatus. Small holes (500 µm diameter) were drilled at a 20° angle relative to the vertical plane above ZIr (AP −1.2 mm, ML ± 2.2 mm, DV −4.4 mm) or PAG (AP −4.4 mm, ML ± 1.5 mm, DV −2.2 mm). The cannulas were lowered to the desired depth and fixed in place using dental cement. In the meantime, a screw for head fixation was mounted on top of the skull with dental cement. Light from a blue LED source (470 nm, 10 mW, Thorlabs) was delivered at a rate of 20 Hz (20-ms pulse duration) via the implanted-cannulas using a bifurcated patch cord (Ø200 µm, 0.22 NA SMA 905, Thorlabs) for ChR2 or GFP control animals. The plastic sleeve (Thorlabs) securing the patch cord and cannula was wrapped with black tape to prevent light leakage. Light from a green LED source (530 nm, 10 mW, Thorlabs) for ArchT animals was delivered in a similar way. Animals were allowed to recover for one week before behavioral tests. During the recovery period, they were habituated to the head fixation on the running plate. The head screw was tightly fit into a metal post while the animal could run freely on a flat rotating plate. Following testing sessions, animals were euthanized and the brain was imaged to verify the specificity of virus expression and the locations of implanted-fibers. Mice with mistargeted injections or misplacement of optic fibers were excluded from data analysis.

### Behavioral tests

Flight response: The test was conducted in a sound-attenuation booth (Gretch-Ken Industries, Inc.). Sound stimulation, LED stimulation and data acquisition software was custom developed in LabVIEW (National Instruments). Each mouse was tested for one session per day which lasted no longer than 2 h. During the behavioral session, the animal was head-fixed and the speed of the running plate was detected with an optical sensor and recorded in real time^53^. A 5-s noise sound at 80 dB sound pressure level (Scan-speaker D2905) was applied to trigger flight response as previously described^20^. The stimulus was repeated for 20 trials per session at an irregular interval ranging from 120 to 180 s. The blue or green LED light (lasting for the entire duration of noise presentation) was randomly co-applied in half of the trials. For testing the gain control modulation, noise at 20, 40, 60, 80, and 100 dB sound pressure level with or without coupled LED stimulation were presented in a randomized order for ten repetitions for each condition. For the LED-only control experiments, LED was given in the same way but without noise stimulation. For the PAG activation experiment, 5-s long LED stimuli with different powers (1, 4, 7, and 10 mW) were applied for ten trials. Each animal was tested for consecutive 3 days and data were averaged across days for each animal.

Conditioned fear response: Mice underwent auditory fear conditioning in a custom made conditioning chamber and tested in a test box in a sound-attenuation booth (Gretch-Ken Industries, Inc.). The conditioning chamber and test box were cleaned with 70% ethanol before and after each session. The bedding material in the test box was replaced before each test session. On the first day, the animals were exposed to five tones (5 kHz tone, 80 dB SPL, duration = 20 s) after 10 min habituation in the test box. On the following day (conditioning), they were exposed to the 20-s 5 kHz tone co-terminated with a 0.75-mA foot shock (5 Hz for 1 s with the duration of each pulse = 100 ms) for five times in the conditioning chamber. For testing conditioned fear response, mice (in ChR2, ArchT and GFP control group respectively) were placed in the test box and given 6 tone presentations in the absence of foot shocks, with half of the trials (in a randomized order) paired with LED stimulation. Inter-trial interval was randomly chosen from a range of 120–240 s (mean = 180 s). For fear extinction, mice were given 10 tone presentations in the absence of foot shocks. A subset of these mice underwent extinction training with pairing optogenectic suppression and tone presentations. For testing extinction recall, mice which had undergone extinction training were put in the same test box on the following day and 5 tone presentations were given. Extinction was also tested on head-fixed animals habituated on the same plate used for recording, with the speed of the plate recorded during the whole process. For silencing mPFC, muscimol (200 nl, 1.5 mM) mixed with Alexa Fluor 488 conjugated Dextran (Life Technologies) was pressure injected into ILA/PL regions right before the extinction test. All the behavioral assays were video recorded, and a blind procedure was implemented for analysis. Fear response was scored as the percentage of time freezing during the 20-s presentation of 5 kHz tone. The freezing of the animal was scored if no movement was detected (except for respiratory movements) for at least 1 s, and the total freezing time during a tone presentation was counted based on the video analysis. Animals were excluded if they failed to exhibit freezing upon the first CS representation one day following the conditioning, as defined by <30% of time freezing.

Open field locomotion test: Mice were placed inside the same test box (25 cm × 25 cm × 50 cm) for testing the baseline locomotion activity. They were allowed to habituate to the arena for 10 min. Each animal was tested for 2 sessions per day and each session lasted 15 min, during which blue or green LED stimulation (5 s On/5 s Off) was applied. The animal’s movements were recorded with an infrared camera mounted on the top center of the arena. The mouse position was determined by using custom made semi-automated MATLAB-based tracking software.

Balance beam test: Mice were trained to walk along a 70-cm long and 2-cm wide beam elevated 30 cm above the bench. The beam was connected to an enclosed goal box. Following the training, the animal was placed at the other end and allowed to pass the beam to reach the goal box. Each animal was tested 6–8 trials per day and blue or green LED applied was applied randomly in half of these trials. In the stimulation trial, the LED stimulation lasted the entire duration of the animal’s walking. The time for the animal to cross the beam was recorded and averaged across trials.

### Slice preparation and recording

To confirm the input and output connectivity of ZIr, GAD2::Ai14 mice injected with AAV2/1-pEF1α-DIO-hChR2-eYFP in ZIr, vGLUT2::Ai14 mice injected with AAV1-Syn-Cre mixed with AAV2/1-pEF1α-DIO-hChR2-eYFP in ZIr, or wild-type C57BL/6 mice injected with AAV1-CamKII-hChR2(E123A)-eYFP-WPRE-hGh in mPFC were used for slice recording. Three weeks following the injections, animals were decapitated following urethane anesthesia and the brain was rapidly removed and immersed in an ice-cold dissection buffer (composition: 60 mM NaCl, 3 mM KCl, 1.25 mM NaH_2_PO_4_, 25 mM NaHCO_3_, 115 mM sucrose, 10 mM glucose, 7 mM MgCl_2_, 0.5 mM CaCl_2_; saturated with 95% O_2_ and 5% CO_2_; pH = 7.4). Coronal slices at 350 µm thickness were sectioned by a vibrating microtome (Leica VT1000s), and recovered for 30 min in a submersion chamber filled with warmed (35 °C) ACSF (composition:119 mM NaCl, 26.2 mM NaHCO_3_, 11 mM glucose, 2.5 mM KCl, 2 mM CaCl_2_, 2 mM MgCl_2_, and 1.2 NaH_2_PO_4_, 2 mM Sodium Pyruvate, 0.5 mM VC). PAG and ZIr neurons surrounded by EYFP^+^ fibers were visualized under a fluorescence microscope (Olympus BX51 WI). Patch pipettes (~4–5 MΩ resistance) filled with a cesium-based internal solution (composition: 125 mM cesium gluconate, 5 mM TEA-Cl, 2 mM NaCl, 2 mM CsCl, 10 mM HEPES, 10 mM EGTA, 4 mM ATP, 0.3 mM GTP, and 10 mM phosphocreatine; pH = 7.25; 290 mOsm) were used for whole-cell recordings. Signals were recorded with an Axopatch 200B amplifier (Molecular Devices) under voltage clamp mode at a holding voltage of –70 mV for excitatory currents or 0 mV for inhibitory currents, filtered at 2 kHz and sampled at 10 kHz^54^. Tetrodotoxin (TTX, 1 μM) and 4-aminopyridine (4-AP, 1 mM) were added to the external solution for recording monosynaptic responses only^55^ to blue light stimulation (10 ms pulse, 3 mW power, 10–30 trials, delivered via a mercury Arc lamp gated with an electronic shutter). Gabazine (4 μM) was added to the external solution to block GABAergic currents.

### In vivo recording in head-fixed animals

One week before electrophysiological recordings, mice were anaesthetized using 1.5% isoflurane and a head post was attached as described previously^56^. For recording during conditioning, the animal was head-fixed on the shock plate and a 16-channel silicon probe (NeuroNexus) was lowered into ZIr. It was exposed to the 20-s 5 kHz tone co-terminated with a 0.75-mA foot shock (5 Hz for 1 s with the duration of each pulse = 100 ms) for five times. Spikes were recorded for the first 19 s after the onset of the tone. For recording during extinction, one day before the recording session, animals went through the auditory fear conditioning as described above. On the day of recording, the animal was head-fixed on the running plate (to which it had been habituated) in a sound-attenuation booth. A parylene-coated tungsten electrode (2 MΩ, FHC) or a 16-channel silicon probe (NeuroNexus) was lowered into ZIr. The animal was exposed to the 5-kHz tone for ten times. Spikes during tone presentations were recorded. For silencing mPFC, muscimol (200 nl, 1.5 mM) mixed with Alexa Fluor 488 conjugated Dextran (or saline) was pressure injected into mPFC right before the recording session. To confirm the silencing effect, a tungsten electrode was lowered into the injection area to record spikes before and after the injection. For recording in PAG, GAD2-Cre mice injected with AAV2/1-pEF1α-DIO-hChR2-eYFP in ZIr and implanted with optic cannula above ZIr were head-fixed on the running plate. Recording with a tungsten electrode was carried out at two sites in dlPAG. The animal was exposed to a 50-ms noise (80 dB SPL) for 50 trials to record the sound-evoked responses. Half of these trials were coupled with 50-ms LED stimulation. To examine effects on spontaneous activity in PAG, 25-ms on, 25-ms off, 500 ms LED stimulation was applied without sound stimulation for 50 repetitions. Signals were amplified (Plexon) and recorded with custom made LabVIEW software. The spike timing was analyzed offline. Recording sites were marked by DiI staining (2 mg/ml). Mice were perfused 4% paraformaldehyde right after the recording session to examine the recording site.

### Data processing

For the flight test, running speed was recorded at 10 Hz sampling rate. The onset latency of flight response was defined by the time point at which running speed exceeded the average baseline speed (measured within the 10-s window preceding the noise onset) by 3 standard deviations of baseline fluctuations. Animals were excluded if they did not show robust flight response at the beginning, as defined by noise-induced speed not exceeding baseline by 3 standard deviations. Running traces were normalized based on the peak speed of flight in the absence of optogenetic manipulation. Peak speed was determined as the maximum running speed after averaging 20 running traces for a session. Travel distance was calculated as the integral of running speed within the 5-s stimulation window. For the open field test, normalized travel distance was calculated as the travel distance during the optogenetic stimulation over the baseline travel distance within the same length of time window.

For in vivo extracellular recording, signals were amplified by a preamp (Plexon) at 30 kHz sampling rate. Spike signals were filtered with a 300–3000 Hz band-pass filter. The nearby four channels of the silicon probe were grouped as tetrodes and semi-automatic spike sorting was performed using the offline sorter of Plexon (Dallas, Texas). Clusters with isolation distance >20 were considered as separate clusters^57^. Spike clusters were classified as single units only if the waveform SNR (Signal Noise Ratio) exceeded 4 (12 dB) and the inter-spike interval was longer than 1.2 ms for >99.5% of the spikes. Spike rate was normalized to the average firing rate of the first five trials for each animal. All data analysis performers were blind to the allocation of the experimental groups. The modulation index was calculated by the average firing rate of last three trails divided by that of the first three trials.

### Statistics

Shapiro–Wilk test was first applied to examine whether samples had a normal distribution. The variance was also tested between control and experimental groups. In the case of a normal distribution, equal variance or non-equal variance two-tailed *t*-test was applied depending on the variance test result. Otherwise, a two-tailed non-parametric test (Wilcoxon signed-rank test or Mann–Whitney *U* test in this study) was applied. Statistical analysis was conducted using SPSS (IBM) and Excel (Microsoft).

### Data availability

The data that support the findings of this study are available from the corresponding author upon reasonable request.

## Electronic supplementary material

Supplementary Information(PDF 13918 kb)

## References

[1] Forel, A. Untersuchungen über die Haubenregion und ihre oberen Verknüpfungen im Gehirne des Menschen und einiger Säugethiere, mit Beiträgen zu den Methoden der Gehirnuntersuchung. In Archiv für Psychiatrie und Nervenkrankheiten, **7**, 393–495 (1877)

[2] Mitrofanis J (2005). Some certainty for the ‘zone of uncertainty’? Exploring the function of the zona incerta. Neuroscience.

[3] Zhang X, van den Pol AN (2017). Rapid binge-like eating and body weight gain driven by zona incerta GABA neuron activation. Science.

[4] Moon HC, Park YS (2017). Reduced GABAergic neuronal activity in zona incerta causes neuropathic pain in a rat sciatic nerve chronic constriction injury model. J. Pain Res..

[5] Liu K (2017). Lhx6-positive GABA-releasing neurons of the zona incerta promote sleep. Nature.

[6] Shammah-Lagnado SJ, Negrao N, Silva BA, Silva JA, Ricardo JA (1985). Afferent connections of the magnocellular reticular formation: a horseradish peroxidase study in the rat. Soc. Neurosci. Abs..

[7] Nicolelis MAL, Chapin JK, Lin RCS (1992). Somatotopic maps within the zona incerta relay parallel GABAergic somatosensory pathways to the neocortex, superior colliculus, and brainstem. Brain Res..

[8] Barthó P, Freund TF, Acsády L (2002). Selective GABAergic innervation of thalamic nuclei from zona incerta. Eur. J. Neurosci..

[9] Watson GDR, Smith JB, Alloway KD (2015). The Zona Incerta regulates communication between the superior colliculus and the posteromedial thalamus: implications for thalamic interactions with the dorsolateral striatum. J. Neurosci..

[10] Perier C, Tremblay L, Feger J, Hirsch EC (2002). Behavioral consequences of bicuculline injection in the subthalamic nucleus and the zona incerta in rat. J. Neurosci..

[11] Urbain N, Deschênes M (2007). Motor cortex gates vibrissal responses in a thalamocortical projection pathway. Neuron.

[12] Liu M (2011). Orexin gene transfer into zona incerta neurons suppresses muscle paralysis in narcoleptic mice. J. Neurosci..

[13] Plaha P, Ben-Shlomo Y, Patel NK, Gill SS (2006). Stimulation of the caudal zona incerta is superior to stimulation of the subthalamic nucleus in improving contralateral parkinsonism. Brain.

[14] Plaha P, Khan S, Gill SS (2008). Bilateral stimulation of the caudal zona incerta nucleus for tremor control. J. Neurol. Neurosurg. Psychiatry.

[15] Blomstedt P, Sandvik U, Tisch S (2010). Deep brain stimulation in the posterior subthalamic area in the treatment of essential tremor. Mov. Disord..

[16] Khan S (2011). Outcomes from stimulation of the caudal zona incerta and pedunculopontine nucleus in patients with Parkinson’s disease. Br. J. Neurosurg..

[17] Ma TP, Johnson JC, Hoskins GA (1997). Organization of the zona incerta in the macaque: an electron microscopic study. Anat. Rec..

[18] Mitrofanis J, Ashkan K, Wallace BA, Benabid AL (2004). Chemoarchitectonic heterogeneities in the primate zona incerta: clinical and functional implications. J. Neurocytol..

[19] Kolmac C, Mitrofanis J (1999). Distribution of various neurochemicals within the zona incerta: an immunocytochemical and histochemical study. Anat. Embryol. (Berl.).

[20] Xiong XR (2015). Auditory cortex controls sound-driven innate defense behaviour through corticofugal projections to inferior colliculus. Nat. Commun..

[21] Tovote P (2016). Midbrain circuits for defensive behaviour. Nature.

[22] Bandler R, Shipley MT (1994). Columnar organization in the midbrain periaqueductal gray: modules for emotional expression?. Trends Neurosci..

[23] Chen J, Kriegstein AR (2015). A GABAergic projection from the zona incerta to cortex promotes cortical neuron development. Science.

[24] Oertel WH, Tappaz ML, Berod A, Mugnaini E (1982). Two-color immunohistochemistry for dopamine and GABA neurons in rat substantia nigra and zona incerta. Brain Res. Bull..

[25] Swanson LW, Sanchez-Watts G, Watts AG (2005). Comparison of melanin-concentrating hormone and hypocretin/orexin mRNA expression patterns in a new parceling scheme of the lateral hypothalamic zone. Neurosci. Lett..

[26] Tamamaki N (2003). Green fluorescent protein expression and colocalization with calretinin, parvalbumin, and somatostatin in the GAD67-GFP knock-in mouse. J. Comp. Neurol..

[27] Grofová I, Ottersen OP, Rinvik E (1978). Mesencephalic and diencephalic afferents to the superior colliculus and periaqueductal gray substance demonstrated by retrograde axonal transport of horseradish peroxidase in the cat. Brain Res..

[28] Beitz AJ (1989). Possible origin of glutamatergic projections to the midbrain periaqueductal gray and deep layer of the superior colliculus of the rat. Brain Res. Bull..

[29] Vianna DML, Brandão ML (2003). Anatomical connections of the periaqueductal gray: specific neural substrates for different kinds of fear. Braz. J. Med. Biol. Res..

[30] Boyden ES, Zhang F, Bamberg E, Nagel G, Deisseroth K (2005). Millisecond-timescale, genetically targeted optical control of neural activity. Nat. Neurosci..

[31] Chow BY (2010). High-performance genetically targetable optical neural silencing by light-driven proton pumps. Nature.

[32] Myers KM, Davis M (2002). Behavioral and neural analysis of extinction. Neuron.

[33] Herry C (2010). Neuronal circuits of fear extinction. Eur. J. Neurosci..

[34] Sierra-Mercado D, Padilla-Coreano N, Quirk GJ (2011). Dissociable roles of prelimbic and infralimbic cortices, ventral hippocampus, and basolateral amygdala in the expression and extinction of conditioned fear. Neuropsychopharmacology.

[35] Peters J, Dieppa-Perea LM, Melendez LM, Quirk GJ (2010). Induction of fear extinction with hippocampal-infralimbic BDNF. Science.

[36] Quirk GJ, Mueller D (2008). Neural mechanisms of extinction learning and retrieval. Neuropsychopharmacology.

[37] Monfils MH, Cowansage KK, Klann E, LeDoux JE (2009). Extinction-reconsolidation boundaries: key to persistent attenuation of fear memories. Science.

[38] Milad MRR, Quirk GJJ (2002). Neurons in medial prefrontal cortex signal memory for fear extinction. Nature.

[39] Laurent V, Westbrook RF (2009). Inactivation of the infralimbic but not the prelimbic cortex impairs consolidation and retrieval of fear extinction. Learn. Mem..

[40] Hurley KM, Herbert H, Moga MM, Saper CB (1991). Efferent projections of the infralimbic cortex of the rat. J. Comp. Neurol..

[41] Mitrofanis J, Mikuletic L (1999). Organisation of the cortical projection to the zona incerta of the thalamus. J. Comp. Neurol..

[42] Tervo DGR (2016). A designer AAV variant permits efficient retrograde access to projection neurons. Neuron.

[43] Paré D, Smith Y, Parent A, Steriade M (1988). Projections of brainstem core cholinergic and non-cholinergic neurons of cat to intralaminar and reticular thalamic nuclei. Neuroscience.

[44] Trageser JC (2004). Reducing the uncertainty: gating of peripheral inputs by zona incerta. J. Neurosci..

[45] Ricardo JA (1981). Efferent connections of the subthalamic region in the rat. II. The zona incerta. Brain Res..

[46] Bernays RL, Heeb L, Cuenod M, Streit P (1988). Afferents to the rat red nucleus studied by means ofd-[3H] aspart ate, [3H]choline and non-selective tracers. Neuroscience.

[47] Fanselow MS (1994). Neural organization of the defensive behavior system responsible for fear. Psychon. Bull. Rev..

[48] LeDoux J (2012). Rethinking the emotional brain. Neuron.

[49] Eilam D, Izhar R, Mort J (2011). Threat detection: behavioral practices in animals and humans. Neurosci. Biobehav. Rev..

[50] Barad M, Gean PW, Lutz B (2006). The role of the amygdala in the extinction of conditioned fear. Biol. Psychiatry.

[51] Ibrahim LA (2016). Cross-modality sharpening of visual cortical processing through layer-1-mediated inhibition and disinhibition. Neuron.

[52] Zingg B (2017). AAV-mediated anterograde transsynaptic tagging: mapping corticocollicular input-defined neural pathways for defense behaviors. Neuron.

[53] Zhou M (2014). Scaling down of balanced excitation and inhibition by active behavioral states in auditory cortex. Nat. Neurosci..

[54] Ji XY (2016). Thalamocortical innervation pattern in mouse auditory and visual cortex: laminar and cell-type specificity. Cereb. Cortex.

[55] Petreanu L, Mao T, Sternson SM, Svoboda K (2009). The subcellular organization of neocortical excitatory connections. Nature.

[56] Liang F (2015). Sensory cortical control of a visually induced arrest behavior via corticotectal projections. Neuron.

[57] Harris KD, Hirase H, Leinekugel X, Henze DA, Buzsáki G (2001). Temporal interaction between single spikes and complex spike bursts in hippocampal pyramidal cells. Neuron.

