# Supplementary material for: Inhibitory gain modulation of defense behaviors by zona incerta
Source: Nat Commun. 2018 Mar 20;9:1151. doi: 10.1038/s41467-018-03581-6 (PMC5861117; doi:10.1038/s41467-018-03581-6)
Supplement: Supplementary file 1 — Supplementary Information(PDF 13918 kb) [file 41467_2018_3581_MOESM1_ESM.pdf]

Inhibitory Gain Modulation of Defense Behaviors by Zona Incerta

Chou et al.

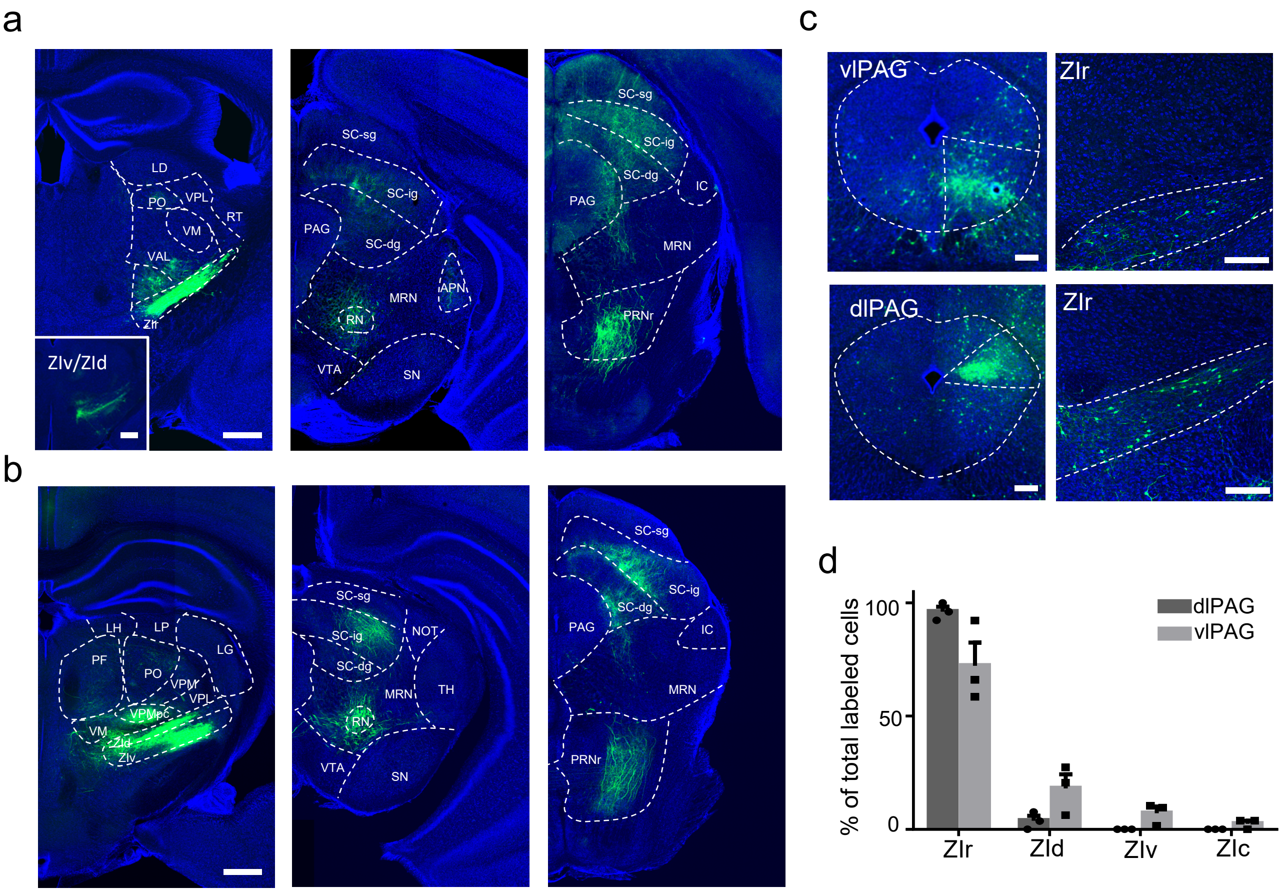

Supplementary Fig. 1. Projection patterns of ZIr and ZIv/ZId.

a, Injection of Cre-dependent GFP virus in ZIr of an example GAD2-Cre mouse. Three panels show GFP expression at different coronal levels. Inset of the left panel, an image at the level of ZIv/ZId; note that florescence signals are mainly from projecting axons instead of labeled cell bodies. Scale bars, 500 µm. **b,** Injection of Cre-dependent GFP virus in ZIv/ZId of an example PV-Cre mouse. Three panels show GFP expression at different coronal levels. Scale bar, 500 µm. **c,** Injection of rabies-ΔG-eGFP into the ventrolateral PAG (vlPAG, upper panel) or dorsolateral PAG (dlPAG, lower panel) of wild-type mice. Left, GFP-labeled neurons around the injection site. Right, retrogradely labeled neurons in ZIr. Blue shows Nissl staining. Scale: 200 µm. **d,** Comparison of relative numbers of labeled neurons (expressed as the percentage of total labeled neurons in ZI) in different ZI subdivisions between dlPAG and vlPAG injections (n = 3 mice for each). Abbreviations: LD, Lateral dorsal nucleus of the thalamus; PO, Posterior complex of the thalamus; RT, Reticular nucleus of the thalamus; VM, Ventral medial nucleus of the thalamus; VAL, Ventral anterior-lateral complex of the thalamus; ZIr, Zona incerta, rostral; LH, Lateral habenula; LP, Lateral posterior nucleus of the thalamus; LG, Lateral geniculate complex; PF, Parafascicular nucleus; VPM, Ventral posteromedial nucleus of the thalamus; VPL, Ventral posterolateral nucleus of the thalamus; VPMpc, Ventral posteromedial nucleus of the thalamus, parvocellular part; ZId, Zona incerta, dorsal; ZIv, Zona incerta, ventral; SC-ig, Superior colliculus, intermediate gray; SC-dg, Superior colliculus, deep gray; SC-sg, Superior colliculus, superficial gray; PAG, Periaqueductal gray; MRN, Midbrain reticular nucleus; APN, Anterior pretectal nucleus; RN, Red nucleus; VTA, Ventral tegmental area; SN, Substantia nigra; TH, Thalamus; NOT, Nucleus of the optic tract; PRNr, Pontine reticular nucleus, rostral; IC, Inferior colliculus. Solid symbol represents mean ± s.e.m. for **d**.

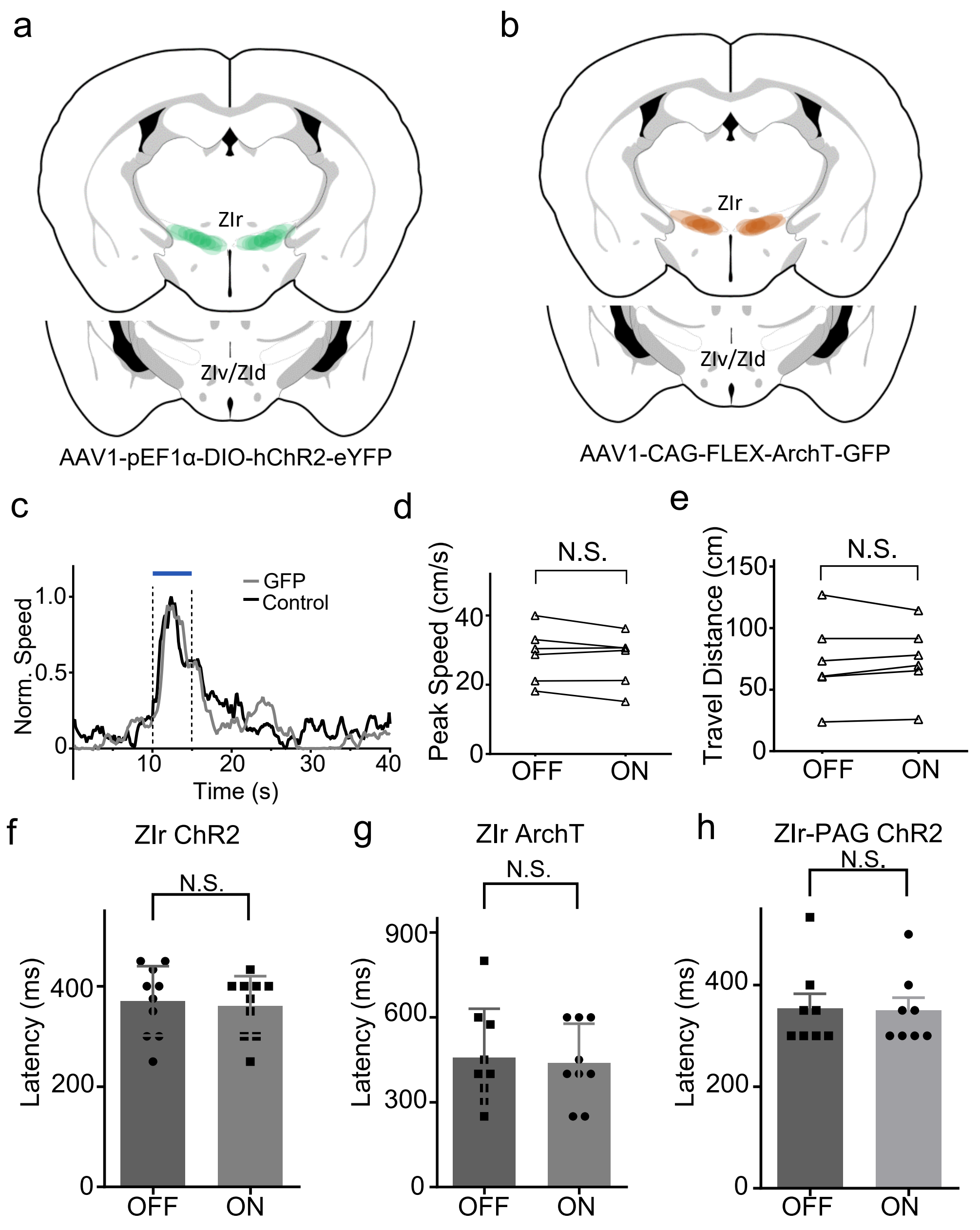

Supplementary Fig. 2. GFP control for LED manipulation and effects of optogenetic stimulation on the latency of noise induced flight.

a & b, Summary of area of ChR2 and ArchT expression in neuronal cell bodies around injection sites (shaded) for two coronal levels (upper, ZIr; lower, ZIv/d). N = 11 animals for ChR2; 9 for AchT injections. **c,** Normalized average speed traces for an example ZIr GFP-expressing animal with and without LED stimulation. Two dash lines mark the duration of noise presentation. Solid bar indicates the duration of LED stimulation (20-ms pulses at 20 Hz). **d,** Summary of noise-induced peak speed with (ON) and without (OFF) LED stimulation for control animals injected with AAV-GFP in ZIr. N.S., not significant, p = 0.196, two-sided paired t-test, n = 6 animals. **e,** Summary of total travel distance during the 5-s noise stimulation with and without LED stimulation for GFP control animals. N.S., not significant, p = 0.687, two-sided Wilcoxon-signed rank test, n = 6 animals. **f & g,** Summary of onset latency of noise induced flight response for ZIr ChR2 and ArchT expressing groups with and without LED stimulation in ZIr. N.S., not significant, p(f) = 0.157, p(g) = 0.683, two-sided paired t-test, n = 10 animals for ChR2; 9 for AchT. **h,** Onset latency of noise induced flight response for the experimental group in which ZIr-PAG axonal terminals were stimulated by LED. p = 0.317, two-sided Wilcoxon-signed rank test, n = 8 animals. Solid symbol represents mean ± s.e.m. for all panels.

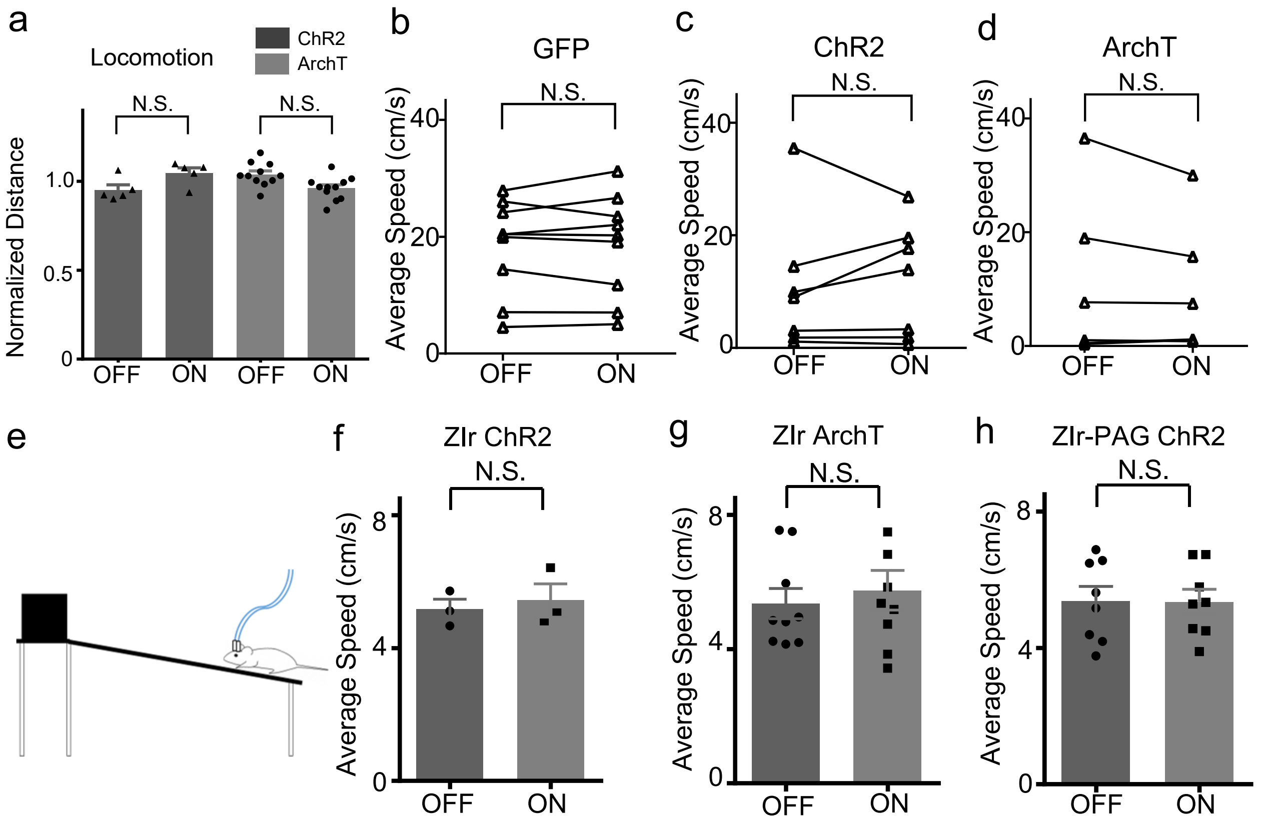

Supplementary Fig. 3. Manipulations of ZIr activity do not affect baseline locomotion and balance beam performance.

a, Normalized travel distance in the open field locomotion test with and without LED stimulation. N.S., non-significant, $p > 0.05$, Paired t-test, Black, ChR2-expressing group (n = 5 animals); gray, ArchT-expressing group (n = 6). **b-d,** Summary of average running speed in the absence of noise with and without LED stimulation for GFP, ChR2 and ArchT injected group respectively. N.S., not significant, p(b) = 0.789, p(c) = 0.556, p(d) = 0.271, two-sided paired t-test, n = 9 animals for GFP, 7 for ChR2, 6 for ArchT group respectively. **e,** Experimental paradigm for balance beam test. A mouse needs to move cross the beam to reach a larger safer platform. **f-h,** Summary of average speed of crossing the balance beam with and without LED stimulation for different experimental groups. N.S., not significant, p(f) = 0.777, p(g) = 0.322, p(h) = 0.846, two-sided paired t-test, n = 11 animals for ZIr ChR2, 9 for ZIr AchT, and 8 for ZIr-PAG ChR2 groups respectively. Solid symbol represents mean $\pm$ s.e.m. for all panels.

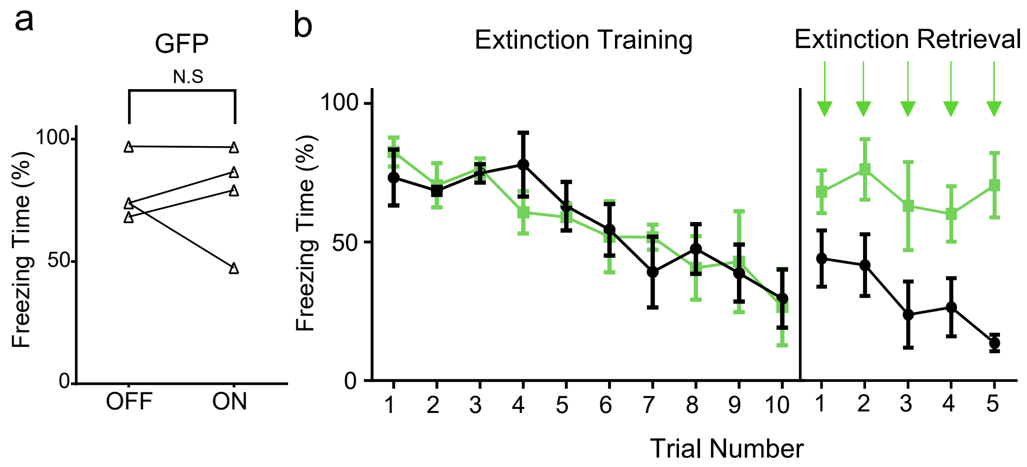

Supplementary Fig. 4. GFP control for conditioned freezing and the effect of inactivation of ZIr on extinction retrieval.

a, Percentage of time freezing during presentation of CS alone with and without LED stimulation in ZIr for GFP expressing control animals. N.S., not significant, $p = 0.939$, two-sided paired t-test, $n = 4$ animals. **b,** Percentage of time freezing during 10 presentations of CS on the day following conditioning (i.e. extinction training) and during 5 presentations of CS (i.e. extinction retrieval) on the day following extinction training. Arrows point to the trials in which LED was applied. $N = 3$ animals.

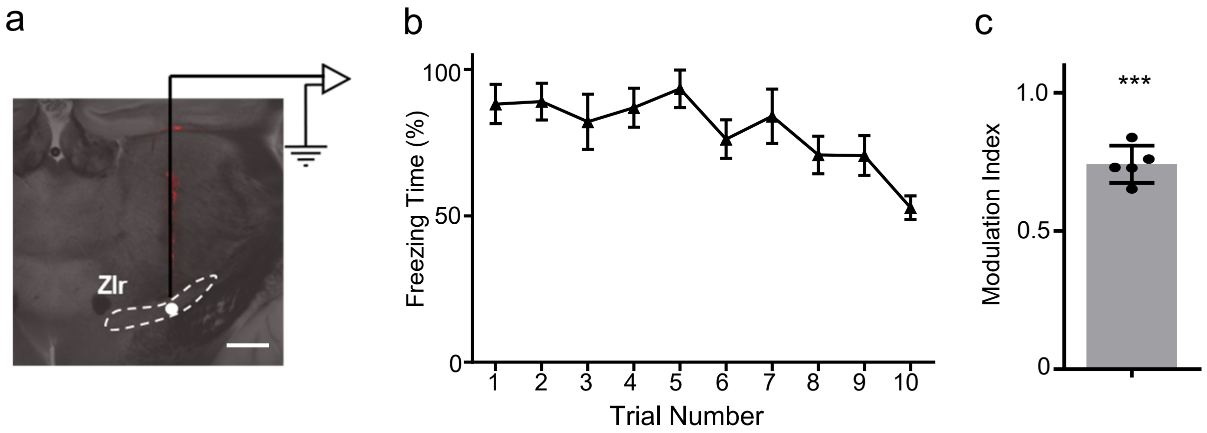

Supplementary Fig. 5: Fear extinction in head-fixed animals.

a, Image showing recording site in ZIr. Scale bar: 500 µm. **b,** Percentage of time freezing during repeated 10 presentations of CS (i.e. extinction training) for head-fixed animals on the recording plate, n = 5 animals. The freezing time decreases with increasing trial numbers similar as in the freely moving condition. **c,** Modulation index for fear extinction in head-fixed animals. The index is significantly smaller than 1, indicating a decrease in freezing time during extinction. ***, $p < 0.001$, two-sided one sample t-test, n = 5 animals. Solid symbol represents mean ± s.e.m. for **b** and **c**.

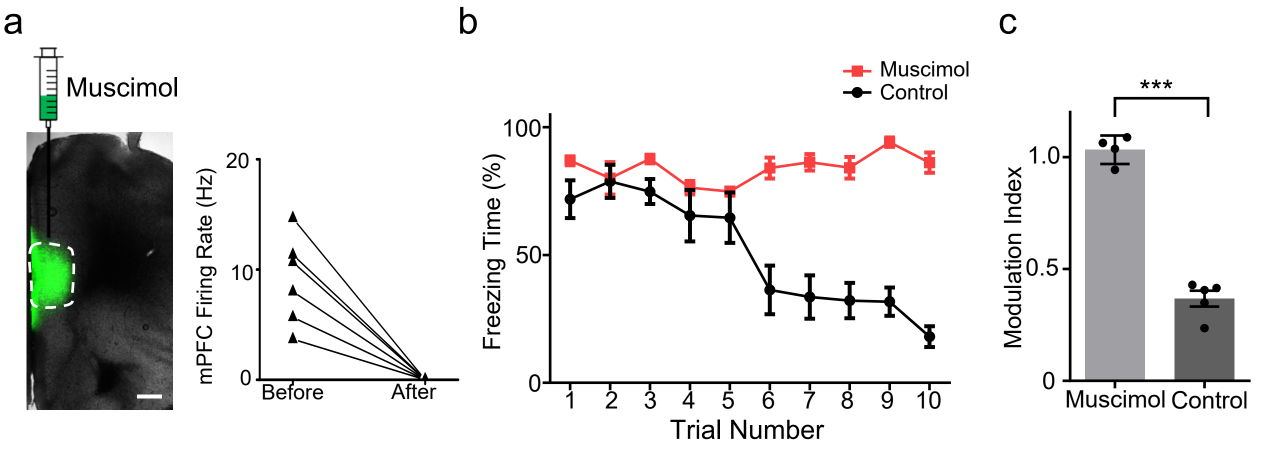

Supplementary Fig. 6. Silencing of the medial prefrontal cortex (mPFC) impairs fear extinction.

a, Left, image showing site for fluorescent muscimol injection in mPFC. Scale bar, 500 µm. Right, multiunit recording in mPFC before and after the muscimol injection, n = 6 animals. **b,** Percentage of time freezing during 10 presentations of CS for control and mPFC muscimol injected animals, n = 4 animals for muscimol and 5 animals for control group. **c,** Modulation index quantified for muscimol and control group. ***, $p < 0.001$, two-sided unpaired t-test, n = 4 animals for muscimol and 5 animals for control group. Solid symbol represents mean ± s.e.m. for **b** and **c**.

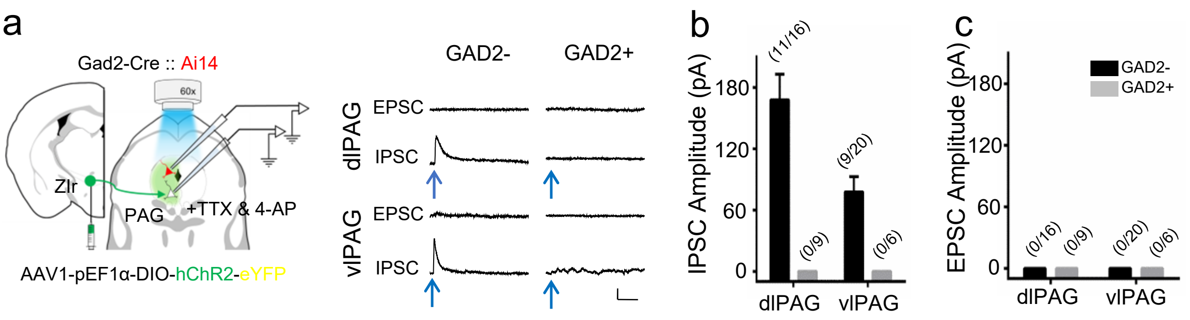

Supplementary Fig. 7. ZIr directly innervates excitatory but not inhibitory neurons in both dlPAG and vlPAG.

a, Left, experimental paradigm: AAV encoding Cre-dependent ChR2 injected into ZIr of GAD2-Cre::Ai14 mice, and whole-cell recording made from excitatory (white, unlabeled) or inhibitory (red, labeled by tdTomato) neurons in PAG. Blue LED was applied to stimulate ZIr axons in PAG. Right, LED evoked excitatory (EPSC) and inhibitory postsynaptic currents (IPSC) in example excitatory and inhibitory neurons in dlPAG and vlPAG respectively. Arrow points to the onset of LED. Scale: 40 pA, 100 ms. **b,** Average amplitudes of LED-evoked IPSCs in excitatory (black) and inhibitory (grey) PAG neurons. Numbers in parentheses indicate the number of neurons exhibiting evoked responses out of the total number of recorded cells. **c,** No EPSC was observed in any of the recorded cells. Solid symbol represents mean ± s.d. for **b.**

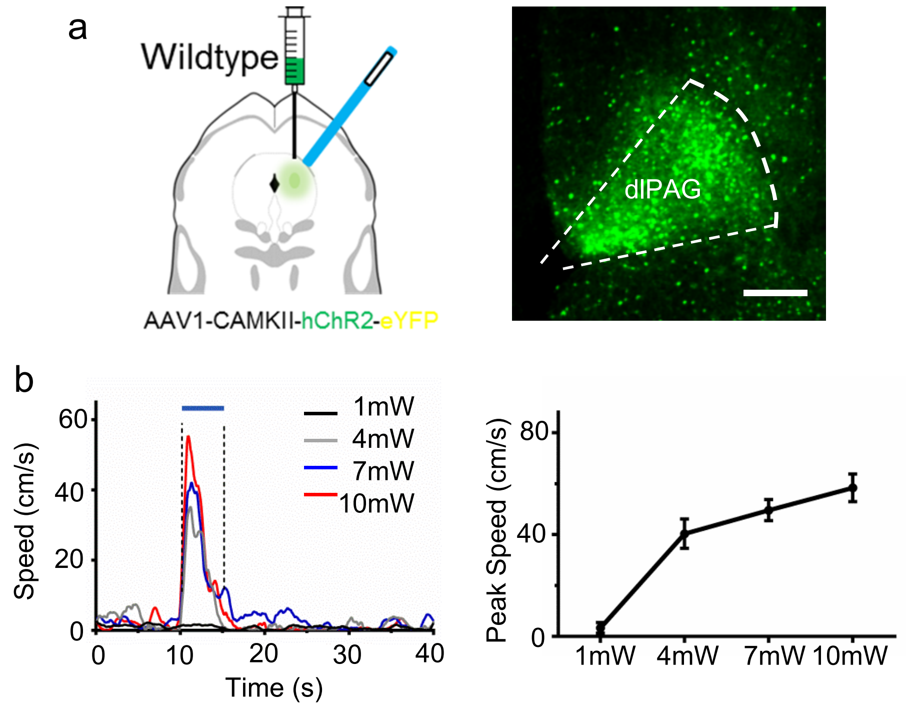

Supplementary Fig. 8. Activation of dlPAG neurons directly induces flight response and its magnitude is dependent on LED intensity.

a, Left, experimental paradigm: ChR2 virus injected into dlPAG and LED stimulation in PAG. Right, an example image of ChR2 expression in PAG. Scale bar, 200 µm. **b,** Left, average speed traces for an example animal under different LED light intensities. Right, summary of average peak running speeds under different LED light intensities, plotted as mean ± s.e.m., n = 3 animals.
